# Surface Physicochemical Properties at the Micro and Nano Length Scales: Role on Bacterial Adhesion and *Xylella fastidiosa* Biofilm Development

**DOI:** 10.1371/journal.pone.0075247

**Published:** 2013-09-20

**Authors:** Gabriela S. Lorite, Richard Janissen, João H. Clerici, Carolina M. Rodrigues, Juarez P. Tomaz, Boris Mizaikoff, Christine Kranz, Alessandra A. de Souza, Mônica A. Cotta

**Affiliations:** 1 Departamento de Física Aplicada, Instituto de Física Gleb Wataghin, Universidade Estadual de Campinas, Campinas, São Paulo, Brazil; 2 Centro APTA Citros Sylvio Moreira, Instituto Agronômico de Campinas, Cordeirópolis, São Paulo, Brazil; 3 Institute of Analytical and Bioanalytical Chemistry, University of Ulm, Ulm, Germany; University of Waterloo, Canada

## Abstract

The phytopathogen *Xylella fastidiosa* grows as a biofilm causing vascular occlusion and consequently nutrient and water stress in different plant hosts by adhesion on xylem vessel surfaces composed of cellulose, hemicellulose, pectin and proteins. Understanding the factors which influence bacterial adhesion and biofilm development is a key issue in identifying mechanisms for preventing biofilm formation in infected plants. In this study, we show that *X. fastidiosa* biofilm development and architecture correlate well with physicochemical surface properties after interaction with the culture medium. Different biotic and abiotic substrates such as silicon (Si) and derivatized cellulose films were studied. Both biofilms and substrates were characterized at the micro- and nanoscale, which corresponds to the actual bacterial cell and membrane/ protein length scales, respectively. Our experimental results clearly indicate that the presence of surfaces with different chemical composition affect *X. fastidiosa* behavior from the point of view of gene expression and adhesion functionality. Bacterial adhesion is facilitated on more hydrophilic surfaces with higher surface potentials; XadA1 adhesin reveals different strengths of interaction on these surfaces. Nonetheless, despite different architectural biofilm geometries and rates of development, the colonization process occurs on all investigated surfaces. Our results univocally support the hypothesis that different adhesion mechanisms are active along the biofilm life cycle representing an adaptation mechanism for variations on the specific xylem vessel composition, which the bacterium encounters within the infected plant.

## Introduction

Biofilms are complex microbial communities associated with surfaces with important biological functions including bacterial infection and enhanced resistance against antimicrobial agents [1]. The bacterial biofilm life cycle is known as a multi-stage process, which current models subdivide into five development stages [2]. During all these stages, biofilm development may be mediated by a number of factors including nutrient environment, pH, temperature and surface properties [2]. Several factors may affect the initial microbial adhesion to such surfaces; these factors include the physicochemical properties of surfaces and cells, and the particular conditions within the environment where adhesion occurs [3]. Moreover, surface properties may be altered by the adsorption of (macro) molecules from the liquid environment onto the substrate forming a conditioning film, which has been considered as the initial step of biofilm formation [3-5]. Advanced understanding the influence of surfaces properties on biofilm formation will therefore lead to strategies for preventing microbial adhesion, and consequently, biofilm development.

Within microbiology research, many previous studies [6-12] have focused on the question which different substrate surface properties - such as roughness, topography, stiffness, hydrophobicity and charge density - play important roles during bacterial adhesion and/or biofilm formation. The variability of results found in literature, however, evidence the importance of experimental conditions in drawing conclusions about the systematic behavior of bacterial cells. Furthermore, surfaces are seldom homogeneous at both micro and nanoscale; such heterogeneities - as in micropatterned surfaces - can influence cell processes such as phagocytosis of filamentous *Escherichia coli* bacteria by macrophages [13]. We can thus expect that cell adhesion will be influenced as well. This is an important issue to the organization of larger structures such as biofilms. Hochbaum and Aizenberg [14] have shown that bacterial ordering and oriented attachment on the single cell level can be induced by nanometer-scale periodic surface features. The surface patterning causes spontaneous and distinct cell arrangement for Gram-positive and Gram-negative bacterial strains and can be modeled by maximizing the bacteria contact area with the surface. The authors suggest that this behavior is a general phenomenon involved in natural biofilm organization.

In our present study, we address the role of surface properties at different length scales on adhesion of the Gram-negative bacteria *Xylella fastidiosa*, which has recently been included in the list of top 10 plant pathogenic bacteria in molecular plant pathology [15]. This microorganism is a xylem-inhabiting bacterium responsible for diseases of economically important crops such as plum, almond, peach, coffee, grapevine and citrus [16,17]; its widely accepted mechanism of pathogenicity is attributed to water and nutrient stress due the blockage of the xylem vessels caused by biofilm formation. Nonetheless, the precise mechanism of *X. fastidiosa* adhesion and biofilm formation on a molecular level is not well understood to date.

Moreover, different *X. fastidiosa* biofilm architectures have been reported for the two main environments where the bacterium is found, i.e., insect cuticle or xylem vessel. In insects, cells adopt a polar configuration regarding the attachment point and form a mat-like structure; in plants, attachment to the xylem wall does not show any preferential orientation [18]. The biofilm architecture is a crucial issue since it may related to the bacterial adhesiveness. In fact, the transmission from host to vector relies on a balance regarding the pathogen adhesiveness: the bacteria should be sufficiently adherent to be acquired by the insect, but not so much as to be withheld within the host [19]. This phenotypic plasticity, which allows bacteria survival in multiple environments, is most likely regulated by signals provided by the habitat.

Despite the importance of the environment in the bacteria life cycle, in vitro studies of *X. fastidiosa* biofilms have been largely performed on abiotic surfaces which are not related to the natural bacterial habitat [20-24]. Cellulose, hemicellulose, pectin and proteins are constituents from xylem vessels. Among the cell wall proteins, extensins and hydroxyproline/proline-rich, which are structural proteins, are known to reinforce the polysaccharide networks [25]. Recently a large number of other cell wall proteins in different organs were found by proteomics approaches ( [26,27], accessible via the public database (www.polebio.scsv.ups-tlse.fr/WallProtDB/)) revealing a great diversity of protein functions in the dynamics of cell walls, which involves organization and rearrangements of polysaccharides in addition to cell-to-cell communication [28]. The plant cell walls forming the xylem can be degraded by enzymes, thus facilitating the spreading of the bacteria, and most likely, providing carbohydrates as an additional nutrient source [20]. In plant studies of *X. fastidiosa* biofilm formation are very difficult to perform due to the dynamics of microbial cell movement and the simultaneous presence of different stages of biofilms within the xylem vessels. Therefore, surfaces containing xylem constituents, which can also have their properties mapped by physicochemical tools at different scales, have not been used on in vitro studies of this bacteria to date. Within this work we use a pool of techniques to study bacteria-surface interactions, as well as its role on biofilm development, at the different length scales of interest to the problem. A comparative study of the main surface properties usually considered in microbiology research, for substrates which simulate the natural bacteria habitat, was thus carried out.

In order to address the role of the surface properties on the adhesion of *X. fastidiosa*, we have used both abiotic (silicon) and biotic (derivatized cellulose) surfaces to study biofilm development. Abiotic surfaces are considered inert materials (e.g. glass, silicon) while biotic surfaces contain substances which can be used as nutrients by the microbial cells. Regarding biotic surface mimicry, we used derivatized cellulose (or cellulose functionalized by chemical modification) thin films to resemble more closely the plant conditions since cellulose represents the major constituent in the natural *X. fastidiosa* habitat. In this context, synthetic cellulose - such as cellulose acetate (CA) and ethyl cellulose (EC) - thin polymer films were used in order to provide supports with biotic character and different chemical functional groups (acetate ester groups and ethyl ether groups, respectively) resulting in electrical charge differences, whereas EC exhibits less negative charge as CA and is comparable to the standard cellulose. The analysis of surface properties was performed using micro- and nanoscale analytical techniques at the relevant bacterial cell and membrane/protein length scales, respectively. Microscopy and gene expression analyses show that the presence of different types of cellulose at the surface influence the adhesion, development rate and architecture of *X. fastidiosa* biofilms. Surface properties like roughness, hydrophobicity/hydrophilicity and surface potential were characterized considering in addition the effect of exposure to the culture medium. Finally, force-distance curves between the transmembrane XadA1 adhesion protein and the investigated surfaces were recorded for determining potential interactions at the initial stages of adhesion. Taken these data altogether, a scenario with relevant parameters controlling bacterial adhesion can be drawn.

## Material and Methods

### Bacteria strain and growth conditions

The *X. fastidiosa* subspecies *pauca* strain used in this study was 9a5c. The strain was grown in Periwinkle Wilt (PW) medium including Bovine Serum Albumin (BSA) as described in previous work [4].

### Preparation of silicon and derivatized cellulose surfaces

Silicon surfaces (<100> oriented, cut into square shapes of approximately 2x2 cm^2^) were rinsed with acetone, 2-propanol and deionized (DI) water to remove the organic contamination from the surfaces. To obtain the cellulose acetate (CA) solution, typically 0.7 g of cellulose acetate powder (Sigma-Aldrich) was dissolved in a solution containing 7.4 ml of acetic acid, 13.8 mL of acetone and 7.4 ml of deionized water. In the ethyl cellulose (EC) case, 0.3 g of ethyl cellulose powder (Sigma-Aldrich) was dissolved in 30 ml ethanol. Both solutions were deposited onto the silicon substrates via spin coating (3000 rpm, 30 s). The samples were dried overnight at room temperature (RT). All surfaces were sterilized via autoclaving procedures.

### Biofilm development

*X. fastidiosa* cells were grown from frozen state to a liquid PW medium for static biofilm development. The cells were incubated simultaneously at 28°C on different autoclaved surfaces for 7, 14, 21 days-cycles without medium replenishing. All samples were prepared under similar conditions and repeated from 2 to 5 times.

### Real-time quantitative polymerase chain reaction (RT-qPCR)

Biofilms were scraped from the different surfaces and the RNA was extracted using RNeasy Mini Kit (Qiagen), following the manufacturer protocol. To RT-qPCR analysis cDNA synthesis was performed using 1 µg total RNA using SuperScriptII Reverse Transcriptase (Life Technologies), and Random Primers oligonucleotide 0,1 mM (Life Technologies), 1x Reaction Buffer (Life Technologies), dNTP 0,1 mM and RNAseOUT RNAse Inhibitor (100 U) (Life Technologies), in a total volume of 20 µl. RT-qPCR was performed in duplicate in an optical 96-well plate with ABI 7500 Fast sequence detection system (Life Technologies), in a volume of 25 µl, containing 2 µl of cDNA, 1X SYBR Green Master Mix (Life Technologies), ROX as passive reference and 200 nM of both reverse and forward primers. The following thermal conditions were used for all PCR reactions: 50°C for 2 min, 95°C for 10 min and 40 cycles of 95°C for 15 s and 60°C for 60 s. The mRNA levels were quantified in relation to the endogenous control gene *petC*. The expression level was presented as 2^-ΔΔCt^, where ΔCt = Ct_target_ – Ct_*pETC*_ and ΔΔCt = ΔCt_treatment_ - ΔCt_Si_. The results of measurements were expressed as mean ± standard deviation (SD). Data were evaluated by one-way analysis of variance (ANOVA) with a significance level of α=0.05 followed by Tukey’s post hoc test.

### Optical and Electron Microscopy

The samples were mapped by Zeiss (*AXIO Scope A1*) optical microscopy to quantify the number and diameter of individual biofilms on the whole sample surface. We used IMAGEJ software to estimate the biofilm diameter assuming circular areas. Scanning electron microscopy (SEM) images were acquired using a dual-beam FIB/SEM system (Quanta 3D FEG, FEI Company, Eindhoven, The Netherlands). The samples were gently water-rinsed (to remove the PW medium and no adherent microbial cells) and were then dried at RT overnight

### Atomic force microscopy (AFM)

Topography images, surface potential and force-distance curves were acquired using an Agilent AFM system Model 5500 (Agilent Technologies, Chandler, AZ, USA). AFM topography images were acquired in tapping mode in air using conical Si tips with a typical tip radius of 10 nm and tip length of ~20 µm (NSC14/AIBS MikroMash, Tallin, Estonia). The spring constant and resonance frequency were typically within the range 1.8-12.5 Nm^-1^ and 110-220 kHz, respectively. To evaluate the roughness of the surface, the root-mean-squared roughness (RMS) was determined over 5x5 µm^2^ areas for each sample. Spectral analysis of the images was carried out by calculating the local maxima for the Power Spectral Density Function. All AFM images were analyzed using freely available software (Gwyddion V. 2.29). SP images were acquired simultaneously with topography in tapping mode under N_2_ flow using Pt-coated Si tips (NSC18/Pt, MikroMash, Tallin, Estonia). In order to observe possible surfaces changes by PW medium, all surfaces were kept in contact with PW medium for 24 h. The samples were then rinsed in DI water and dried at RT overnight. In the Amplitude Mode-Kelvin Probe Force Microscopy technique used here, we define the ‘zero’ SP value for the first point of the image; thus only variations of SP along the image can be compared quantitatively. Therefore, in order to compare SP values from different surfaces and thus exclude tip effects arising from metal coating wear [29] we have used the Si substrate as a common reference in the images; for that we have scanned edges of the cellulose film which show the Si substrate as well. We thus compare absolute SP* values from the different samples according to the following equation: SP* = SP(cellulose) – SP(Si). Each SP* value was obtained by averaging five cross sections per image across the edge between homogeneous regions of cellulose film and silicon. To evaluate SP variation along the surface, the root-mean-squared SP roughness (SP-RMS) was determined over 5x5 µm^2^ areas for each sample similarly to the procedure carried out for topography images. In order to assess the electrical properties of the PW film, we have removed it to form a step on the Si surface. The same procedure for SP* evaluation was followed to the PW film - in this case SP* = SP(PW film) - SP (Si).

### *XadA1* immobilization on AFM tip and force-distance curves

Conical Si tips (NSC19/AIBS MikroMash, Tallin, Estonia) with spring constant typically within the range 0.17-1.7 Nm^-1^ were cleaned in piranha solution (H_2_SO_4_:H_2_O_2_ = 3:1) for 5 min and rinsed with running DI water. The cantilevers were subsequently incubated in 3-(glycidoxypropyl) trimethoxysilane (GOPTES) solution for 30 min; cantilevers were then rinsed with ethanol and DI water and dried under N_2_-flow. To immobilize *XadA1* on the tip, the cantilevers were immersed in 15 µm/ml *XadA1* in PBS (pH 7.4) buffer for 30 min and washed in TBS-TT buffer (20 mM Tris/HCl, 500 mM NaCl, 0.5% (v/v) Tween 20, 0.2% (v/v) Triton-X100, pH 7.4) and TBS buffer (10 mM Tris/HCl, 150 mM NaCl, pH 7.4) for 5 min each. Finally, the tips were rinsed with PBS buffer for 1 min and stored in the same buffer solution. The successful immobilization of the *XadA1* adhesion protein on the tip was subsequently confirmed by fluorescence microscopy (Figure **S1**) via specific polyclonal *XadA1* antibody [30] and fluorescein labeled anti-rabbit IgG second antibody (Rheabiotech, Campinas, Brazil). Details of the experimental method to obtain purified *XadA1* protein and antibody were published previously [51]. All force-distance curves were obtained using liquid cell buffered with PBS (pH 7.4) solution or PW medium (control experiment) at RT. Cantilevers were moved at a velocity of 200 nm/s with total z-displacement of ~700 nm. Data were acquired ~200 times at each of five different locations on each surface. For the CA and EC surfaces, control experiments included force-distance curves acquired on Si surfaces immediately before and after the measurements on the derivatized cellulose surfaces. Control experiments were also carried out with non-functionalized tips for all the investigated surfaces for both PBS and PW medium. Spring constants were experimentally obtained after tip-functionalization using thermal noise method. All adhesion peaks in the retracting force-distance curves were determined in order to build force histograms for each surface. For this statistical analysis, we estimated the noise level of the measurement using over 2500 force-distance curves. Thus, adhesion forces lower than the estimated noise (9±5 pN) or absence of adhesion peaks were both considered as null force (e.g. 0 nN). The average adhesion forces and deviation were calculated using Gaussian fitting to the histogram data using the commercial software Origin 8.0 (OriginLabs, USA).

### Surface contact angle measurements

These measurements were performed as described in a previous work [4]. Samples exposed to PW medium for different time intervals were all washed with DI water and dried at room temperature overnight. Contact angles lower than 10° were considered as complete wetting.

## Results and Discussion

### Experimental approach

The *X. fastidiosa* subspecies *pauca* strain 9a5c was used within this study. The strain was grown in Periwinkle Wilt (PW) medium [31] including Bovine Serum Albumin (BSA) as described in a previous work [4]. *X. fastidiosa* cells were incubated at 28°C for several days on cellulose acetate (CA) and ethyl cellulose (EC) as well as bare, clean silicon surfaces, for static biofilm development.

Biofilm development was assessed from optical and scanning electron microscopy (OM and SEM, respectively) images of samples dried overnight. Real-time quantitative polymerase chain reaction (RT-qPCR) was used to evaluate specific gene expression changes at different biofilm development stages. In order to probe changes in the hydrophilic character of the different substrates used here, contact angle measurements were performed for all three surface compositions. Substrate surface properties were also mainly investigated using Atomic and Kelvin Probe Force Microscopy (AFM and KPFM, respectively), as well as force spectroscopy measurements with XadA1 adhesin-functionalized silicon tips. In particular, KPFM [32,33] allows topography and surface potential (SP) imaging of the sample, within a nanometer range spatial resolution. The contact potential between tip and sample is defined by the difference between their work functions, Δϕ [34,35]. By applying external bias voltages and compensating the contact potential, KPFM cancels this particular electrostatic interaction between tip and sample and makes it possible to measure SP = Δϕ/e, where *e* represents the electron charge, simultaneously with the sample topography. KPFM has been used extensively as a unique method to characterize the nano-scale electronic/electrical properties of metal/semiconductor surfaces, semiconductor devices and more recently, organic materials/devices and biological materials [36]. Although an important variable to adhesion models, SP values are not usually determined in biological processes, and the zeta potential of the surface is used instead. Zeta potential is an electrokinetic method, which provides the potential difference between the dispersion medium and the stationary fluid of layer attached to a dispersed particle. Such measurements on cellulosic materials have been extensively studied in literature [37]; for CA and EC surfaces values range in the -10 to -50mV [38,39], depending on various parameters such as pH, polymer swelling, fiber structure, etc. However, zeta potential measurements are not suitable for surface mapping or relating potential and topography inomogeneities. Therefore, we have elected a direct measurement of SP (in dry atmosphere) to compare such inomogeneities in our samples. However, it is important to notice that, once immersed in liquid, the surface electrostatic properties can be altered by the presence of ions in the surrounding solution, due to chemical adsorption or electrical double layer formation. Screening of such inomogeneities can occur in solution; this issue will be further addressed in the subsequent sections.

### *X. fastidiosa* biofilm architecture and development rate dependence on surface properties

**Figure 1:**
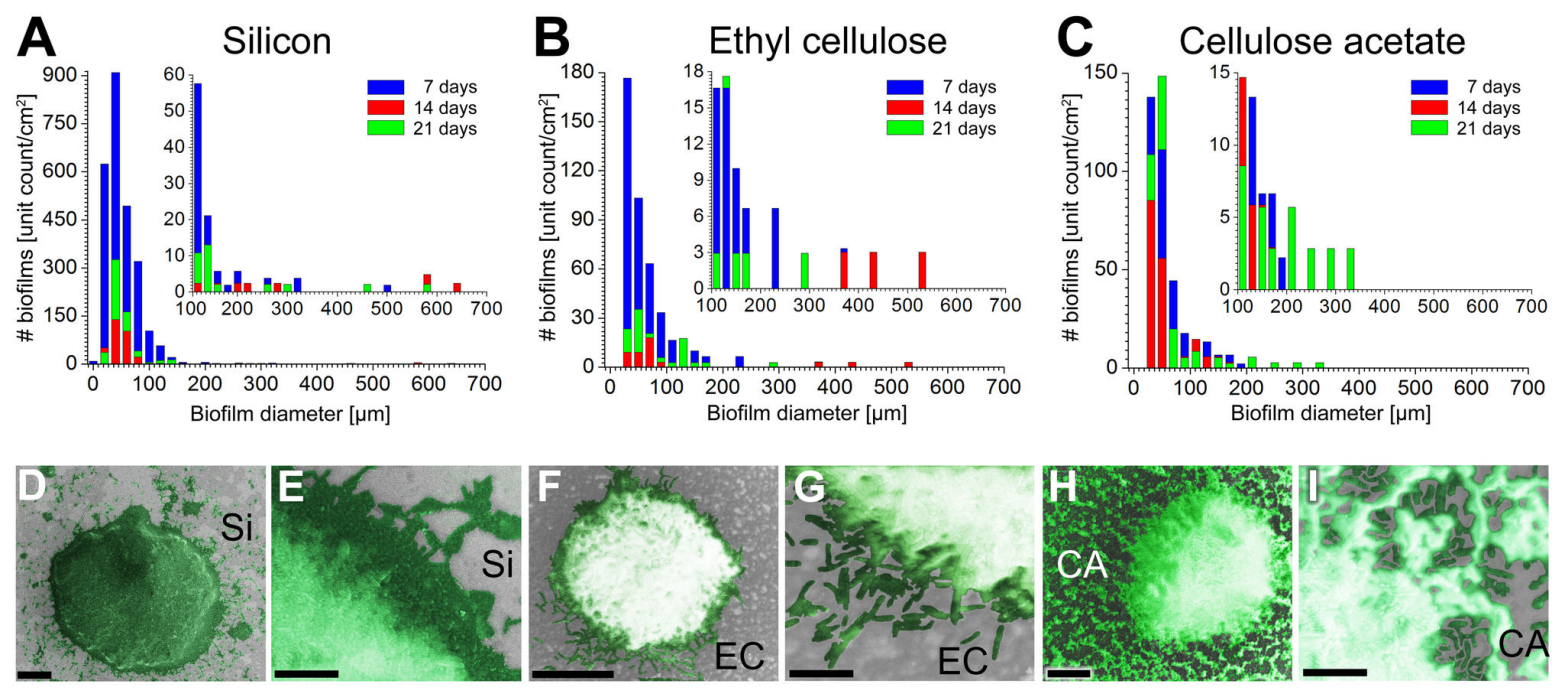
Biofilm architecture and quantitative biofilm size distribution. Size distribution histograms (**A**-**C**) and color enhanced SEM images of biofilms architecture (**D**-**I**) grown on bare silicon (Si; **A, D, E**), ethyl cellulose (EC; **B, F**, **G**) and cellulose acetate (CA; **C, H, I**) substrates in PW medium. Images E, G and I show details of the biofilm edges on each substrate at higher magnification. Scale bars correspond to 20µm for D, F, H and 5µm for **E**, **G**, **I**. The insets in **A-C** show re-scaled histograms to more precisely visualize the presence of larger biofilms.

**Figure 2:**
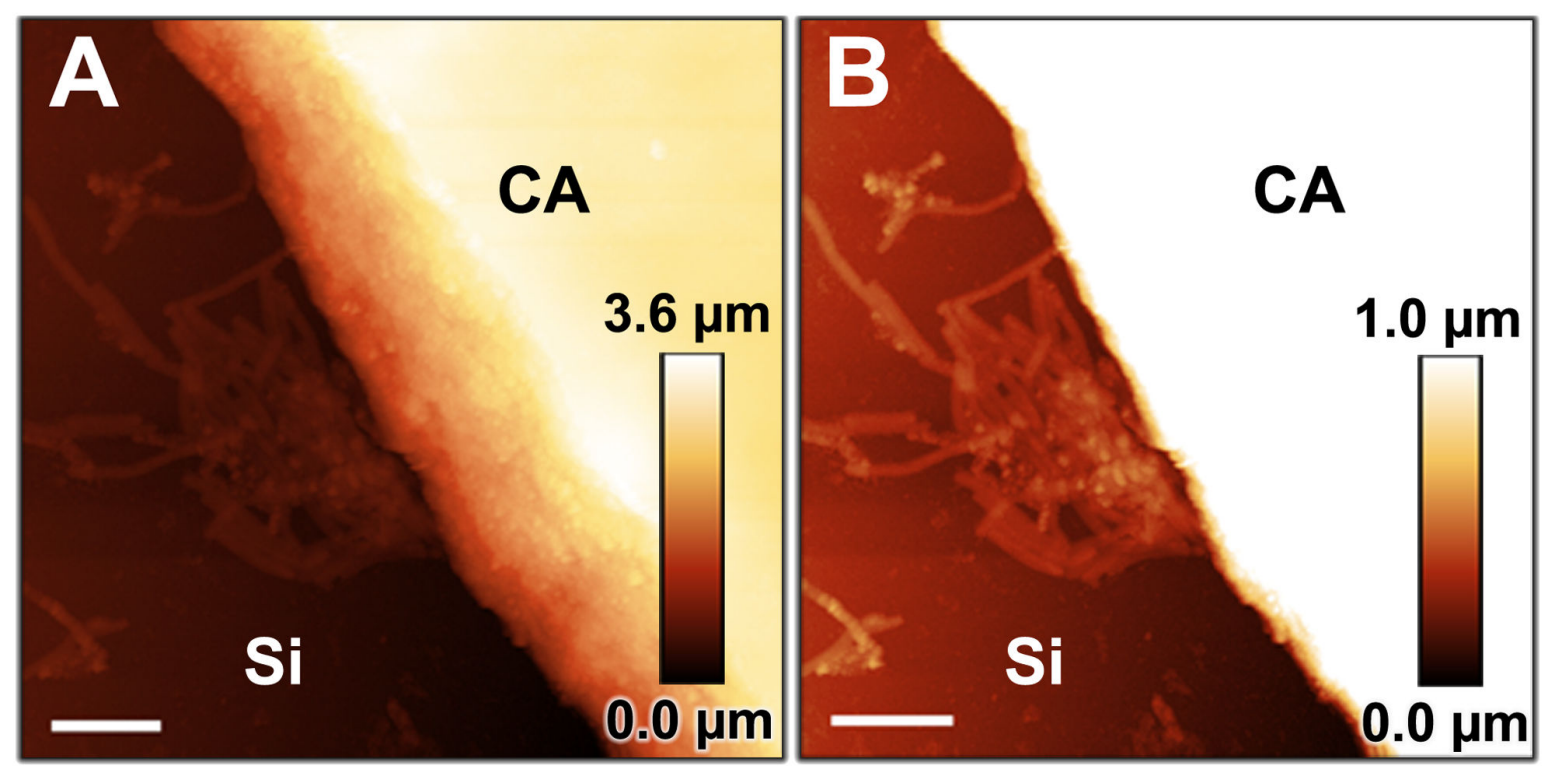
Comparison of biofilm growth on pristine silicon and cellulose acetate surfaces. AFM topography images of *Xylella fastidiosa* biofilms grown on bare silicon (Si) and cellulose acetate (CA) surfaces side-by-side in periwinkle wilt (PW) medium (scale bar 5 µm). Images are shown with different height contrasts to illustrate individually the CA topography (**A**) and biofilm (**B**) more accurately. As a control image, please refer to Figure **S2A** in supplemental information.

Both development and architecture of *X. fastidiosa* biofilms were compared at silicon (abiotic, stiff) and derivatized cellulose (biotic, soft) surfaces. Figure **1** shows the size distribution and typical architecture for biofilms on Si, EC and CA. In good agreement with previous studies [4,23], the biofilms typically present circular shapes with a different range of diameters along a 21-day cycle for all investigated surfaces (Figure **1 A-D, F, H**); no ridges or wrinkles were observed during the entire period [40]. Considering the same growth time, Si surfaces present a larger number of biofilms (approx. 5-fold) compared to derivatized cellulose surfaces. Despite the dispersion of diameter values, there is a clear predominance of smaller biofilm structures (approximately 20-80µm in diameter) on all surfaces after 7 and 21-day growth cycles (Figure **1 A-C**). This expected behavior is attributed to the initial and last stages of the life cycle of *X. fastidiosa* biofilms, as e.g., the detachment of bacteria from the biofilms provides nucleation centers for new sites [41]. For 14-day growth periods, the number of small biofilm structures along Si and EC surfaces decreases significantly independent of the surface material, while the biofilm diameter increases (Figure **1 A, B**). This behavior can be interpreted as the result of a competition process, i.e., some biofilms grow in size at the expenses of the smaller ones due to nutrient consumption [23,41] or simply due to the spatial overlap of the biofilms. In contrast, although the total number of 14-day biofilms on CA also decreases, they do not present significant changes in diameter distribution until they are 21 days old (Figure **1 C**). This analysis also reveals larger biofilm diameters on Si surfaces; for example, the largest biofilm observed on CA surfaces after 21 days (approximately 350 µm diameter) is only half the size of the largest biofilm structure observed on Si surfaces after only 14 days (approximately 650 µm diameter). Contrarily to CA samples, biofilms on EC surfaces also reach larger diameters after only 14 days (approximately 550 µm diameter). Interestingly, despite a similar overall shape, the edges of the biofilms vary significantly according to the substrate. SEM images reveal a more extensive first layer and well-defined edges for *X. fastidiosa* biofilms on Si and EC surfaces (Figure **1 E, G**); in fact, particularly on Si, the first layer usually extends well beyond the more massive region of the biofilm for all growth times considered in the present experiments. For CA surfaces, however, the biofilm edges are not well defined; a random distribution of groups of cells is observed in the region around the biofilms, but usually disconnected from the large matrix deposit (Figure **1 I**). In fact, even for older (21 days) samples, the regions surrounding the edges of the biofilms are not entirely filled with cells, as in the case of Si and EC surfaces, i.e., patches of exposed substrate can be found among the bacteria surrounding the biofilms on CA surfaces. Furthermore, if Si and CA surfaces are exposed side-by-side to bacteria, biofilm formation occurs earlier on Si than on CA, as shown by corresponding AFM experiments (Figure **2**). These results provide a first indication that enhanced adhesion and higher development rates for *X. fastidiosa* biofilm growth are prevalent on bare Si surface compared to EC and CA surfaces, respectively, and agree with previous studies which report enhanced adhesion on stiffer substrates [10,42]. Moreover, these results imply that cell-surface interaction strongly depends on the surface properties, which may also affect the biofilm architecture at the microscale during later bacterial life cycle stages.

### Investigation of silicon and derivatized cellulose surface properties

**Figure 3:**
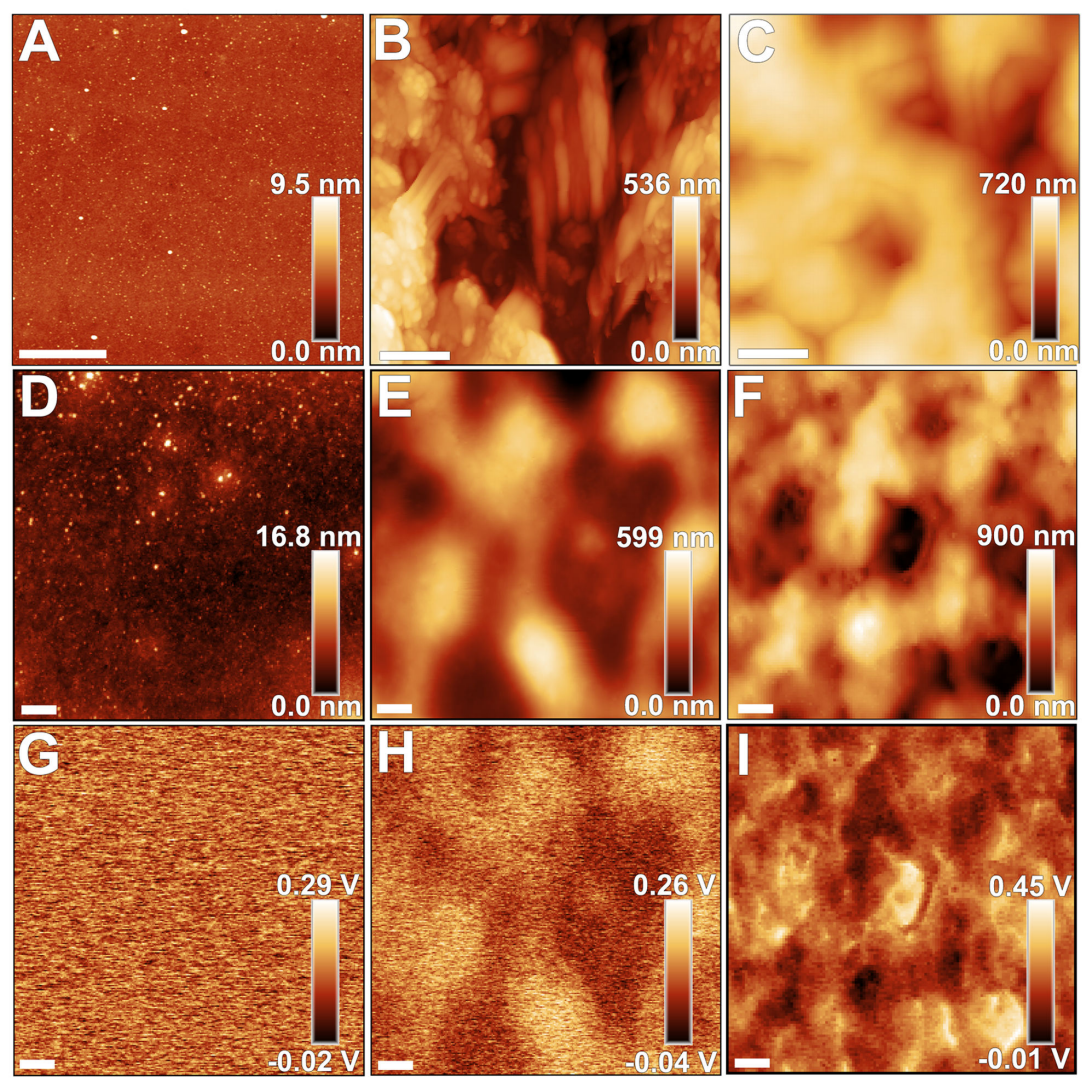
Surface morphology and potential alterations of silicon and cellulose surfaces induced by culture media. AFM topography (**A**-**F**) and surface potential (SP; **G-I**) images of the substrate surfaces. (**A**-**C**) show topography of pristine silicon (Si), ethyl cellulose (EC) and cellulose acetate (CA) surfaces, respectively; (**D**-**F**) illustrates topography of Si, EC and CA surfaces after incubation in PW medium for 24h, respectively; (**G**-**I**) present SP images of the surfaces shown in (**D**-**F**). Scale bars correspond to 1µm.

Surface properties in general can also be modified upon interaction with the culture medium [3-5]. In order to assess the role of such effect on bacterial adhesion and biofilm formation, AFM and contact angle analysis techniques were applied to characterize the investigated surfaces before and after contact with PW medium. AFM topography images reveal different morphologies for each pristine surface (exemplary shown in Figure **3**). As expected, the Si surface presents a flat morphology, while the derivatized cellulose films provide an irregular surface structure. CA samples present granular morphologies homogeneously distributed along the surface (Figure **3**), whereas both granular and fiber morphologies were observed for EC derivatized films (Figure **3**). As a consequence of the different morphologies, these surfaces present distinct RMS roughness values (typically 0.5 nm for Si; 140±40 nm and 110±6 nm for EC and CA surfaces, respectively). This scenario changes upon exposure to PW medium, which emulates the surface encountered by bacteria upon adhesion in our experiments. Despite larger RMS values (approximately 1.5 nm), the Si surfaces before and after exposure remain highly planar (Figure **3**) and homogeneous; in contrast, the PW conditioning film deposited on EC and CA cellulose samples presents opposite characteristics. In the first case, the fiber and grain-like morphologies are buried by the conditioning film, although many features still protrude from the smooth surface due to the initial height variation on the EC surface. Consequently, the RMS values (80±30 nm) are diminished on coated EC surfaces (Figure **3**). On the other hand, CA surfaces present more prominent features than the initial morphology and larger (by approximately 60%) RMS values (180±50 nm). Furthermore, spectral analysis of the obtained topography images for CA surfaces shows that the dominant spatial frequency decreases by a factor of approximately 2, if the conditioning film is present (i.e., from 3.8 to 1.9 µm^-1^).

These results altogether indicate that the conditioning film is not homogeneously deposited on CA surfaces; the deposition does not precisely follow the topography of the initial film, due to the changes observed in both RMS roughness and dominant spatial frequency for the CA surface exposed to PW medium. This behavior suggests that the conditioning film does not purely result from the physical deposition of chemical species within the PW medium on CA surfaces. To further investigate this aspect, we measured the surface potential (SP) on Si and derivatized cellulose surfaces. For the latter, SP variations were observed (Figure **S2**) indicating an inhomogeneous charge distribution at the micron-range length scale, particularly when the fiber morphology is present on the EC sample.

**Figure 4:**
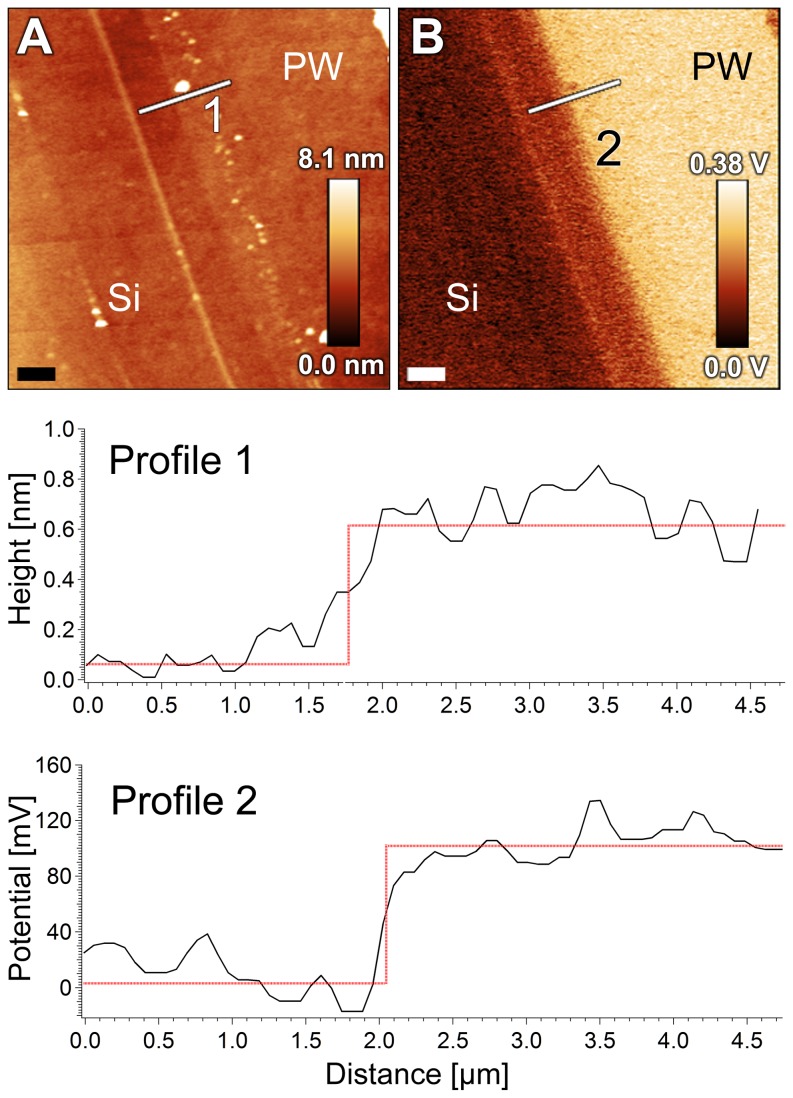
Surface potential of culture media conditioning film formed on pristine silicon. AFM topography (**A**) and surface potential (**B**) images showing the edge of the thin conditioning film caused by periwinkle wilt (PW) medium on silicon (Si) substrate (scale bar 2 µm). The corresponding profiles of height (1) and surface potential differences (2) are shown below.

After interaction with PW medium, however, opposite behaviors are observed for the cellulose films: while SP-RMS diminishes for EC surfaces as the smoothening effect of PW film deposition takes place, SP-RMS increases for the CA surface, and shows dominant spatial frequencies similar to those evident in the corresponding topography images (Figure **3**). Despite these opposite trends, the SP-RMS values after PW exposure are in the same range for both EC and CA surfaces (typically 50-70 mV), although electrically charged and irregularly-shaped micron size domains are more evident on CA surfaces. Moreover, both derivatized cellulose surfaces show smaller average SP values (Figure **S3**) regarding the nearby Si reference, which has also been exposed to PW; the SP inhomogeneities are still present on the surface, as indicated by the darker patches close to the cellulose film edge (Figure **S3**). The difference in surface potential between derivatized cellulose and the nearby Si surface (SP*) depends on the type of cellulose used. Average SP* values obtained for EC and CA were (-150±10) mV and (-200±40) mV, respectively. The effect of the PW film on the electrostatic properties of the surface was evaluated after locally removing the film to form a step structure, and thus, exposing the Si surface underneath; Figure **4** shows that even a very thin PW film (approximately 1 nm thick) introduces a large variation in surface potential (approximately 200 mV).

The variations in surface potential observed along the derivatized cellulose surfaces may also affect their hydrophilic character, which was evaluated using contact angle experiments. Despite the different morphologies and RMS roughness, Si and derivatized cellulose surfaces without previous contact with PW medium present similar degrees of hydrophilicity (Table **1**). Upon contact with PW medium, Si surfaces change from exhibiting moderate (53°) to complete wetting (<10°) properties after only three hours of exposure, while no significant changes were observed for CA surfaces during these experiments. In contrast, EC surfaces have their hydrophilic character enhanced over a longer period of 24 h. These results are better understood by taking into account the different morphologies described above. Stiff and flat surfaces such as Si contribute to more compact conditioning film formation, as previously reported [4]. Thus, the exposed surface for contact angle experiments in this case is mainly composed of this film. On the other hand, for EC and CA surfaces PW medium may take longer to form a continuous conditioning layer; although almost as stiff as Si (as evaluated from force-distance curves using a Si tip in PBS medium, Figure **S4**, which shows a ~15% difference in stiffness), these surfaces present pores and a more irregular morphology which may hinder or delay a continuous coverage. Furthermore, surface inhomogeneities - such as surface roughness - may additionally affect the contact angle, and thus, the surface wetting properties [43], as observed for the CA surface.

**Table 1:** Contact angle variation as a function of exposure time to PW broth for the three surfaces analyzed in this study.

	**Contact angle** [°]		
**Time in contact with PW browth**	**Si surface**	**EC surface**	**CA surface**
w/o contact	53±6	69±4	63±8
3 h	< 10	26±1	63±1
6 h	< 10	32±1	45±1
12 h	< 10	30±1	70±1
24 h	< 10	21±1	67±1

Our results indicate that surface roughness may not be considered an independent variable in *X. fastidiosa* adhesion and biofilm development. In fact, how surface roughness actually affects adhesion at a fundamental level is still under discussion [44,45]. Liu et al. [44] investigated theoretically and experimentally the role of surface roughness during adhesion. In this case, the experimental adhesion studies were performed on silicon surfaces with different degrees of roughness using AFM cantilevers. In contrast to bacterial adhesion and biofilm literature concerning surface roughness [8,12], the authors conclude that the adhesion force decreases as surface roughness increases for a determined feature size. This is indeed observed in our studies, as the largest biofilm structures have been observed on the very flat Si surface. Despite the fact that the physical models for adhesion discussed above do not consider the role of surface chemical species or biomolecules such as e.g., adhesins, our results show that roughness is not a determinant feature for *X. fastidiosa* adhesion, as this process is not inhibited on any of the surfaces studied herein, which show distinctly different roughness values.

Regarding the hydrophobicity/hydrophilicity of the surfaces, hydrophobic termination has been reported as favorable for bacterial adhesion [6,7], but surface properties after interaction with the culture medium are not usually considered in most reports. Adsorption of molecules from the surrounding solution at the substrate surface may change its properties analogously to the natural process of conditioning film formation. In a recent study [4], we reported variations in physicochemical properties of abiotic surfaces due to the conditioning film formed by PW medium constituents and their effect on *X. fastidiosa* biofilm formation. Moreover, attached bacterial cells and biofilms are only observed after time intervals (approximately 4 h) expected for changes in hydrophilicity on Si substrates associated with PW medium effects [46]. Considering these effects observed on all surfaces here, our findings suggest that hydrophobic surfaces do not present favorable conditions for *X. fastidiosa* biofilm formation. Additionally, the extension of the first layer beyond the biofilm deposit (Figure **1 E, G, I**) is larger for more hydrophilic surfaces, thus suggesting that cell-surface interaction is favored in this case. Furthermore, the larger diameter of biofilms grown on CA surfaces after 21 days may result from a more continuous surface coverage with PW film expected from longer growth times [4].

In addition to roughness and hydrophobicity, electrostatic interactions can also enhance initial bacteria adhesion to surfaces [7,22] but only few reports have addressed the role of the surface potential on such processes [7]. Our experimental results show strong evidence of charge distribution changes at abiotic and biotic surfaces due to PW culture medium effect. The Derjaguin-Landau-Verwey-Overbeek (DLVO) theory has been used to explain adhesion of microorganisms to different interfaces [47-49]. This theory considers that the initial cell adhesion results from van der Waals attractive forces and from the repulsive interactions from overlap between the electrical double layer of cell and substrate, whose magnitudes are dependent on the surface potentials. Although our measurements neglect ion shielding and other dynamical, electrical effects arising from surface contact to solution, the SP* values and SP-RMS obtained under N_2_ flow are significantly larger than expected from zeta potential measurements [37-39]. Therefore, electrical gradients due to the micron-sized electrically charged domains on the surface of the cellulose substrates may still be present whenever these are immersed in solution. The interaction potential between this surface and the bacteria membrane should depend on both surface potentials, according to the DLVO theory [50], and thus, the charged domains should most certainly affect the mechanisms *X. fastidiosa* relies upon for adhesion. These data, coupled with our findings of biofilm development, indicate that higher surface potential values and electrically more uniform surfaces provide more favorable conditions for *X. fastidiosa* biofilm formation. Indeed, the micron-sized electrical inhomogeneities present on the cellulose samples are roughly in the length scale of a bacterial cell. Assuming a negatively charged cell membrane for Gram-negative bacteria, adhesion will be certainly affected under such circumstances.

### *XadA1* protein adhesion characteristics on different surface compositions

**Figure 5:**
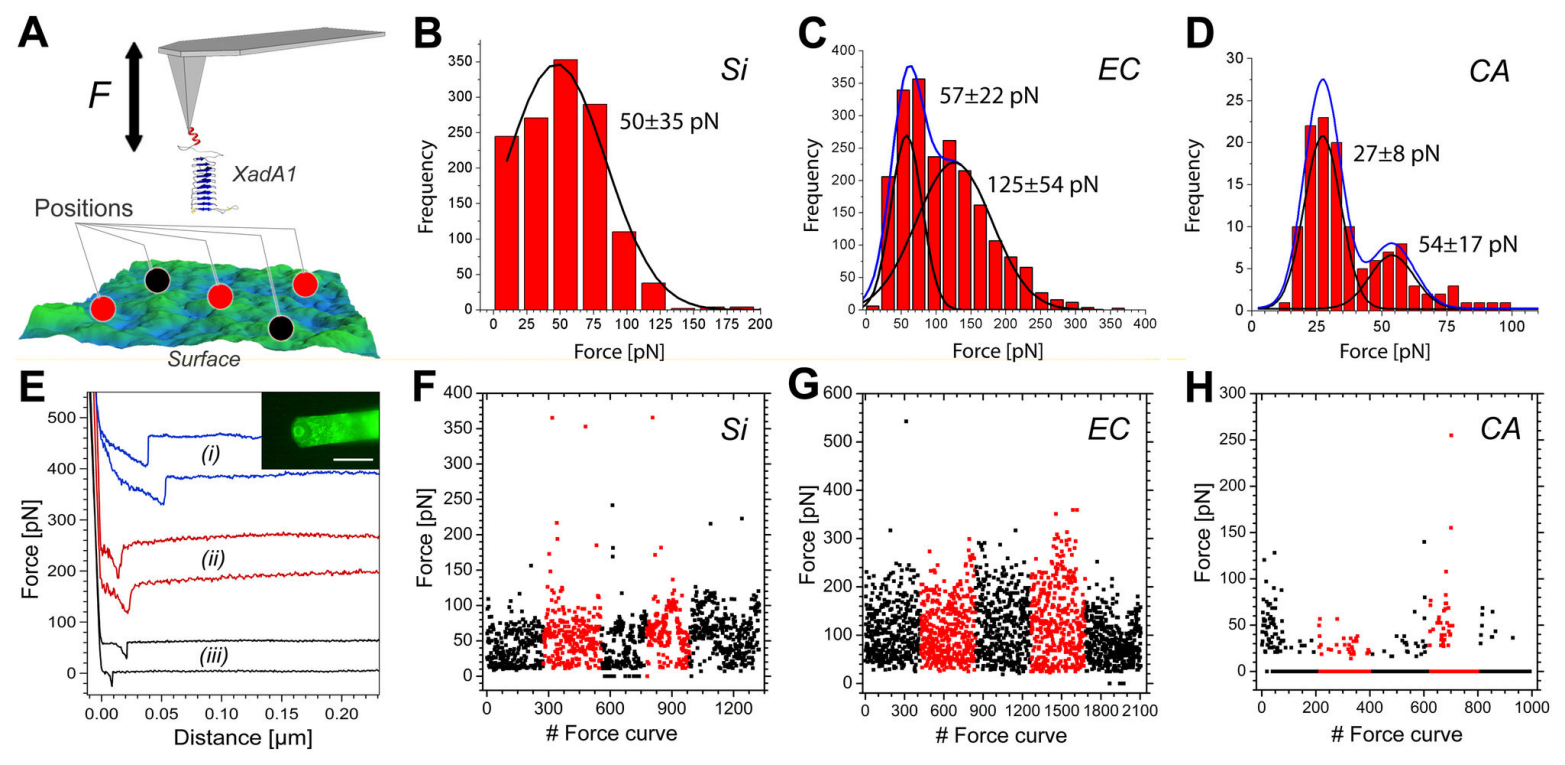
Interaction forces of bacterial adhesin XadA1 on silicon and cellulose surfaces. Schematics of the force spectroscopy (**A**) measurements at five different positions on the surface where force curves were acquired. The force histograms (**B**-**D**) for bare silicon (Si, B) ethyl cellulose (EC, **C**) and cellulose acetate (CA, **D**), show the total number of events (please notice the different scales for the number of events); multiple rupture events were observed for EC and CA. Typical retraction force-distance curves for AFM tips coated with XadA1 adhesion protein (**E**) for Si (i), EC (ii) and CA (iii). The inset shows the fluorescence image of the functionalized AFM tip and cantilever visualized using fluorophore labeled second antibodies (scale bar = 40µm). Measured interaction forces (**F**-**H**) between XadA1 coated AFM tip and bare Si (**F**), EC (**G**) and CA (**H**) substrates in PBS buffer shown in sequential acquisition order. The colors indicate a different region of the sample probed in each set of force curves. The bin sizes for (**B**), (**C**) were adjusted to 22pN and (**D**) to 5pN to allow accurate visualization of the molecular interaction characteristics.

Several reports have shown the important role of fimbrial and afimbrial adhesins in *X. fastidiosa* attachment to surfaces and to each other [51,52]. In this particular case, XadA1 adhesin (ORF XF1516) was recently reported as a relevant protein at all stages of biofilm formation [52]. In order to evaluate how this adhesion protein interacts with surfaces of different composition and homogeneity, we have measured the adhesion forces between XadA1-functionalized AFM Si tips (Figure **S1**) and the substrate surfaces using AFM force spectroscopy (Figure **5 A, E**). The control experiments clearly indicate adhesion peaks also between the surfaces and non-functionalized AFM tips when the PW medium was used (Figure **S5**). This result reveals that the proteins and peptides within the culture medium may promote non-specific adhesion, a role that has been indeed suggested for BSA [53]. Therefore, in an effort to isolate the interaction between XadA1 and the surface from non-specific interactions, all force-distance curves were obtained in PBS buffer (pH 7.4), which did not present any adhesion peaks independent of the surface during control experiments (Figure **S6**).

When recording AFM tip-sample approach curves under these conditions, no adhesion peaks due to van der Waals attractive forces were observed likely due to shielding effects from the surrounding ions within the buffer medium. For the retraction curves with XadA1-functionalized AFM tips, Si and EC surfaces present similar results with a high percentage of adhesion events (Table **2** and Figure **5 B, C**). For both cases, the adhesion forces are in the range of 50-60 pN. Multiples of these values (Figure **5 C, D**) were also found, particularly on the rough cellulose surface, thereby indicating multiple rupture events. Figure **5 (F, G)** shows that the measured forces are homogeneously distributed along the several regions of the sample probed in the experiment for both Si and EC surfaces. Contrarily, the interaction between XadA1 and CA surfaces (Figure **5 D**, Table **2**) occurs at a much lower percentage and also with significantly lower adhesion forces (approximately 30 pN). Furthermore, these events were localized at specific sample regions probed during these studies; some of the CA regions show basically null adhesion forces (Figure **5 H**).

**Table 2:** Average adhesion forces and percentage of single adhesion events from force spectroscopy experiments at the different surfaces analyzed in this study.

**Surface**	**Adhesion force [pN**]	**Adhesion events [%**]
Silicon	50±35	97
Ethyl cellulose	57±22	99
Cellulose acetate	27±8	20

Therefore, from the molecular point of view, the interaction with the specific adhesin XadA1 is reduced for CA surfaces in absence of the PW conditioning film. From the previous analysis of the surface roughness and surface potential on CA surfaces exposed to PW, as well as contact angle measurements, it is hypothesized that the PW film coverage is not continuous on CA surfaces. This implies that the initial CA surface is also exposed upon bacteria adhesion, thus inhibiting the role of XadA1 at many locations of these substrates, as suggested by the spatial distribution of adhesion events reflected in our ensemble of force curves (Figure **5 H**). This hypothesis might also explain the difference in biofilm edges regarding the two derivatized cellulose films (Figure **1 G, I**) if we assume that XadA1 is implicated in the initial adhesion to the surface. However, it is important to notice that the adhesion process of *X. fastidiosa* to CA surfaces may also occur via different adhesins (not investigated in this study) or alternative mechanisms such as e.g., the production of extracellular polymeric substances (EPS) [46] or via carbohydrate-binding proteins [5]. Our force spectroscopy control experiments have shown that proteins such as BSA may also enhance adhesion; similarly to the cell membrane, the surface of the non-functionalized Si AFM tip may exhibit negative charge densities due to the presence of the native oxide [54]. Thus, it is emphasized that although *X. fastidiosa* adhesion and biofilm development did not appear enhanced on CA surfaces as compared to Si, biofilm formation still occurs. These results are in agreement with different colonization rates observed in plant [55].

This conclusion also supports the hypothesis that different bacterial adhesion mechanisms may be active along the biofilm life cycle influencing the biofilm architecture, such as those observed in the transmission vector (insect) and plant xylem [18]. Additionally, no shape transitions or morphogenesis, such as those reported for *Candida albicans* [56], are observed for *X. fastidiosa* in all samples analyzed here. We cannot preclude, however, changes in adhesin density and localization at the cell membrane activated by habitat cues.

### Endoglucanases gene expression dependence on the surface properties

**Figure 6:**
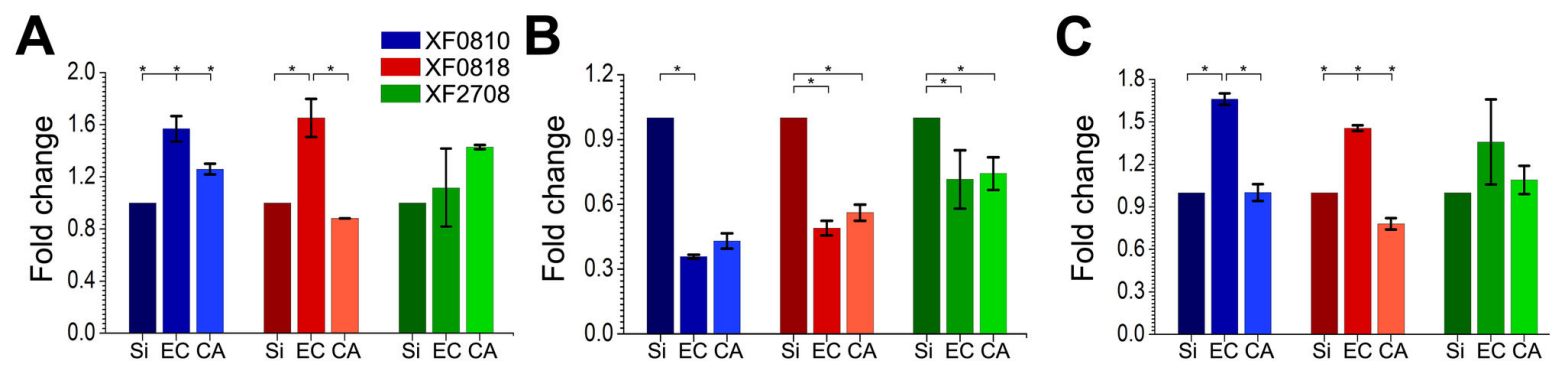
Endoglucanase gene expression dependence on surface composition. Gene expression fold change for the three endoglucanases genes analyzed in the present study for (**A**) 7, (**B**) 14 and (**C**) 21 days of biofilm growth on bare silicon (Si), ethyl cellulose (EC) and cellulose acetate (CA) surfaces. One-way ANOVA with Tukey’s post hoc test was applied in (**A**), (**B**), (**C**). Asterisks denote significance level of α=0.05. Bars represent ± standard deviation.

Our observations show different biofilm development rates and architecture also between the two soft, electrically-inhomogeneous cellulose substrates used here. In fact, Killiny et al. [20] have shown that host structural polysaccharides mediate gene regulation in *X. fastidiosa*; this regulation creates the necessary phenotypic changes for vector transmission. Gene expression analysis also evidences the difference between the two types of cellulose substrates in our study. *X. fastidiosa* has genes encoding endoglucanases enzymes which may degrade cell wall constituents; the expression of these genes could thus be affected by the presence of different celluloses on the surface where the cells attach. The expression of three genes (Table **S1**) related with host cell wall degradation (XF0810, XF0818 and XF2708, http://aeg.lbi.ic.unicamp.br/xf/) was analyzed by qRT-PCR for bacteria grown on derivatized cellulose, in comparison to silicon. Figure **6** shows the gene expression behavior for each derivatized cellulose surface, using the biofilms grown on Si as reference. The expression of these genes was induced after 7 and 21 days especially on EC as surface, and was repressed after 14 days of biofilm formation. This gene expression pattern suggests a negative feedback mechanism arising from the enzyme level present in the immediate environment.

The expression behavior of the genes involved in host cell wall degradation reveals an up-and-down regulation behavior during the biofilm formation, particularly when grown on EC as biotic surface. This behavior may result from a homeostasis strategy to regulate the proteins/enzymes levels required for cellular maintenance [57]. Figure **6** also shows a distinct difference between gene expressions on derivatized cellulose surfaces. Particularly for XF0818, the expression is always enhanced - at 7 and 21 days - if the bacteria are cultivated on the EC surface as compared to CA. *E. coli* clones, which express XF0818, also exhibited cellulose activity and efficiently degraded cellulose [58]. If the rate of degradation varies with the substrate - due to the different chemical structures of EC and CA - so does the carbohydrate amount available to the bacteria, which consequently affects cell metabolism, biofilm formation and biofilm development rate. This process should be facilitated on the EC surfaces also due to the larger contact area between the biofilm and the substrate, as compared to CA surfaces.

These observations clearly indicate the important role of surface properties inside the plant or host on *X. fastidiosa* development. Furthermore, the homogeneity of these properties at micro and nano length scales as well as the effect of conditioning films formed due to the necessary experimental conditions for bacteria survival and proliferation should be considered as well. Particularly, from the biological point of view, the dependence of protein expression and interaction on surface properties suggests the existence of an adaptation mechanism for variations on the specific xylem vessel and sap compositions, which the bacterium encounters within infected plants. In general, our results demonstrate that the surface plays an important role in biofilm formation of *X. fastidiosa*. Moreover, surface properties can be changed by the surrounding solution. Therefore, we suggest that the management strategy to control this phytopathogen should consider the use of compounds such as soil nutritional treatments that could change xylem sap properties and consequently xylem surface characteristics to impair the bacterial colonization.

## Conclusion

In summary, our work shows that stiffer and electrically more homogeneous surfaces with larger surface potential exhibit enhanced *X. fastidiosa* adhesion and proliferation, likely due to a stronger cell-surface interaction under these circumstances. Changes in biofilm architecture and development - such as the colonies sizes and morphological edge features - are attributed to the different physicochemical properties of the biotic and abiotic surfaces studied here, particularly to the inhomogeneities at the microscale, which include the effect of adsorbed species and the conditioning film formed due to the culture medium. The main adhesion protein used by *X. fastidiosa*, XadA1, presents similar interactions forces for both Si and EC surfaces; for CA, however, we observed inhibited XadA1 interaction and a spatial dependence on the regions probed by force spectroscopy. Changes in the gene expression for endoglucanases are also observed depending on the type of surface where adhesion takes place; these changes suggest the influence of the habitat on the bacterial cell metabolism as well. Moreover, habitat modification can interfere on the bacterial colonization.

### Supporting Information

Additional supporting figures and tables are provided in the online available supplemental information.

## Supporting Information

Figure S1 **Epifluorescence images for non-functionalized silicon AFM tip (A) and XadA1 coated AFM tip (B).** For the XadA1 immobilization proof, the coated AFM tips were incubated to a specific anti-rabbit IgG antibody for XadA1 and visualized using a fluorescein labeled anti-rabbit IgG second antibody. Images were acquired with an inverted Nikon Eclipse TE2000U microscope and a photon-counting EMCCD camera (IXON3, Andor, Ireland).
(TIF)

Figure S2 **AFM topography (A, B) and surface potential (C, D) images of cellulose acetate (A, C) and ethyl cellulose (B, D) thin films (scale bar 1 µm).**
(TIF)

Figure S3 **AFM topography (A, B) and surface potential (C, D) images of cellulose acetate (CA; A, C) and ethyl cellulose (EC; B, D) thin film step edges on silicon (Si) substrates after incubation in periwinkle wilt (PW) medium (scale bar 2 µm).**
(TIF)

Figure S4 **Force-distance curves acquired on the three substrates studied, using Si tips measured in PBS medium.** The stiffness values of the plotted linear fits (dashed lines) are shown in the corresponding colors.
(TIF)

Figure S5 **AFM force histogram of a non-functionalized AFM probe on bare silicon (Si) in periwinkle wilt (PW) medium.** The inset shows a zoom-in to illustrate the force distribution in more detail including a Gaussian fitting (black curve).
(TIF)

Figure S6 **Typical approach (red) and retraction (blue) force-distance curves of non-functionalized AFM probes on bare silicon (Si; i), ethyl cellulose (EC; ii) and cellulose acetate (CA; iii) in PBS buffer.**
(TIF)

Table S1 ***Xylella fastidiosa* gene sequences encoding enzymes that may degrade cell wall components, such as cellulose.**
(DOCX)
