# Supplementary figures and images for: Surface Physicochemical Properties at the Micro and Nano Length Scales: Role on Bacterial Adhesion and Xylella fastidiosa Biofilm Development
Source: PLoS One. 2013 Sep 20;8(9):e75247. doi: 10.1371/journal.pone.0075247 (PMC3779164; doi:10.1371/journal.pone.0075247)

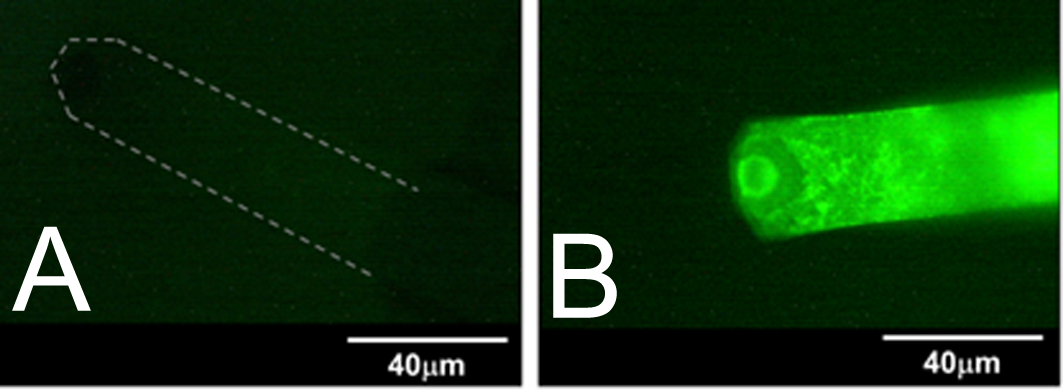

Supplement: Figure S1 — Epifluorescence images for non-functionalized silicon AFM tip (A) and XadA1 coated AFM tip (B). For the XadA1 immobilization proof, the coated AFM tips were incubated to a specific anti-rabbit IgG antibody for XadA1 and visualized using a fluorescein labeled anti-rabbit IgG second antibody. Images were acquired with an inverted Nikon Eclipse TE2000U microscope and a photon-counting EMCCD camera (IXON3, Andor, Ireland). (TIF) [file pone.0075247.s001.tif]

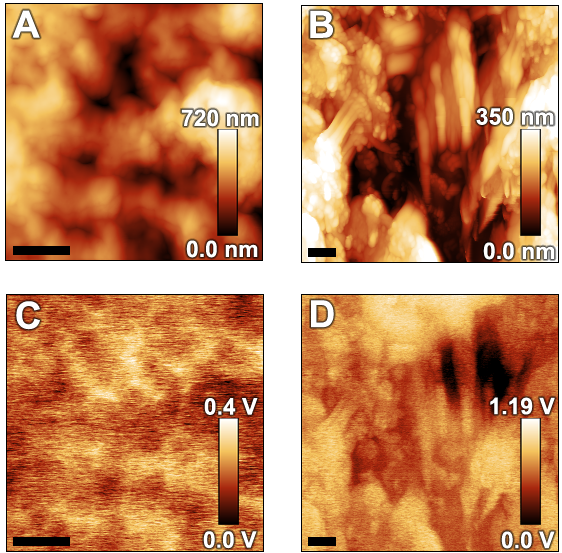

Supplement: Figure S2 — AFM topography (A, B) and surface potential (C, D) images of cellulose acetate (A, C) and ethyl cellulose (B, D) thin films (scale bar 1 µm). (TIF) [file pone.0075247.s002.tif]

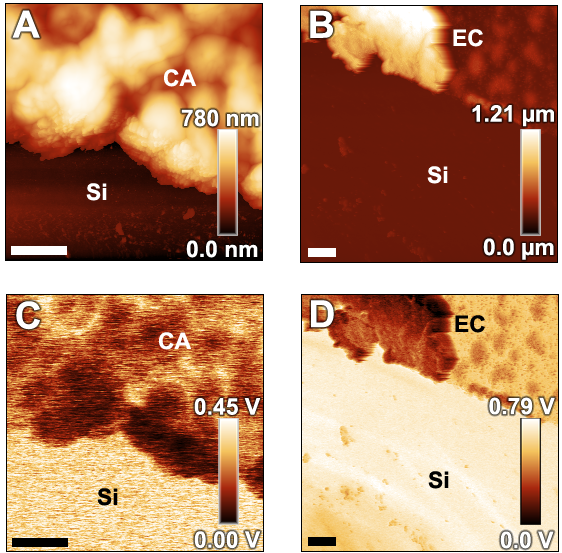

Supplement: Figure S3 — AFM topography (A, B) and surface potential (C, D) images of cellulose acetate (CA; A, C) and ethyl cellulose (EC; B, D) thin film step edges on silicon (Si) substrates after incubation in periwinkle wilt (PW) medium (scale bar 2 µm). (TIF) [file pone.0075247.s003.tif]

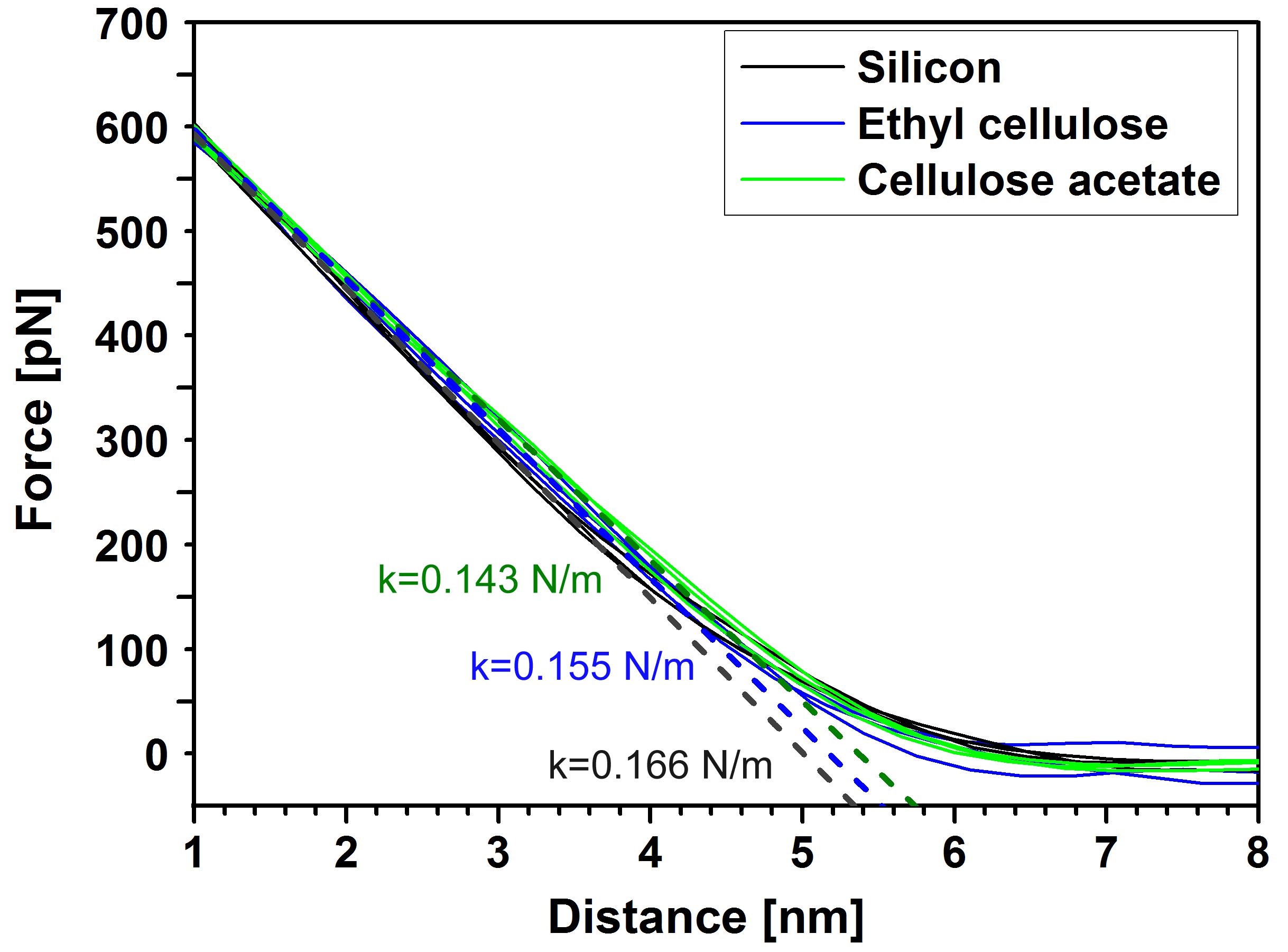

Supplement: Figure S4 — Force-distance curves acquired on the three substrates studied, using Si tips measured in PBS medium. The stiffness values of the plotted linear fits (dashed lines) are shown in the corresponding colors. (TIF) [file pone.0075247.s004.tif]

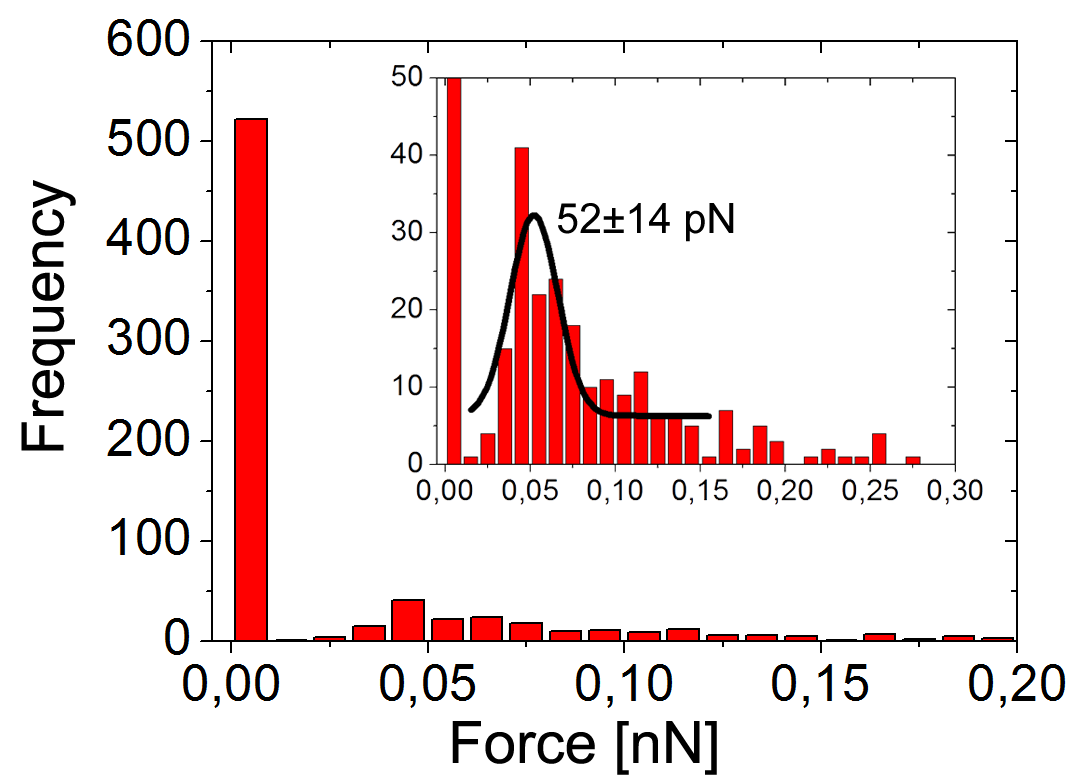

Supplement: Figure S5 — AFM force histogram of a non-functionalized AFM probe on bare silicon (Si) in periwinkle wilt (PW) medium. The inset shows a zoom-in to illustrate the force distribution in more detail including a Gaussian fitting (black curve). (TIF) [file pone.0075247.s005.tif]

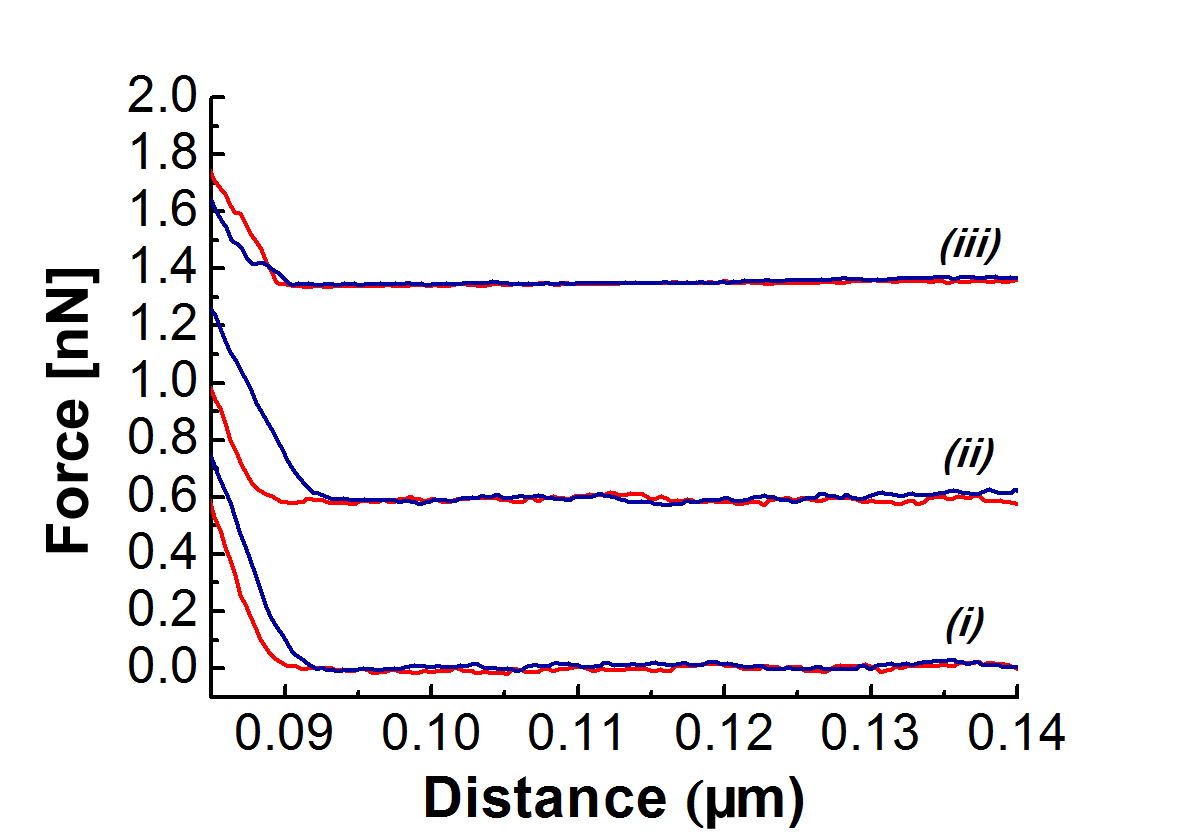

Supplement: Figure S6 — Typical approach (red) and retraction (blue) force-distance curves of non-functionalized AFM probes on bare silicon (Si; i), ethyl cellulose (EC; ii) and cellulose acetate (CA; iii) in PBS buffer. (TIF) [file pone.0075247.s006.tif]
