# Supplementary material for: Surface Physicochemical Properties at the Micro and Nano Length Scales: Role on Bacterial Adhesion and Xylella fastidiosa Biofilm Development
Source: PLoS One. 2013 Sep 20;8(9):e75247. doi: 10.1371/journal.pone.0075247 (PMC3779164; doi:10.1371/journal.pone.0075247)
Supplement: Table S1 — Xylella fastidiosa gene sequences encoding enzymes that may degrade cell wall components, such as cellulose. (DOCX) [file pone.0075247.s007.docx]

**Table S1:** Xylella fastidiosa gene sequences encoding enzymes that may degrade cell wall components, such as cellulose.

| **Gene** | **Primer sequences** | **Fragment size** |
| --- | --- | --- |
| XF0810 | 5´-CATTGTGTCCCGTTACGCAT-3´  5´-CAAGCGCAGCTTCAGTATCA-3´ | 88bp |
| XF0818 | 5´-GATGTCTACGTACAGCCGTA-3´  5´-GTAGCCGGCTTTCGCAAAAT-3´ | 99bp |
| XF2708 | 5´-CTTACGTGGTCAAAAAGGGC-3´  5´-CGTCCAGTGCATCATTCAGT-3´ | 87bp |
| petC | 5´-TCCAGCCAGGTCAGCAGAAC-3´  5´-ACCAAAAAAGTCAACAACACTAGGAA-3´ | 151bp |
